# Solitary infantile myofibromatosis of the extremities: a multicenter case series with descriptive analysis of recurrence and β-catenin expression

**DOI:** 10.1186/s13023-026-04392-5

**Published:** 2026-06-04

**Authors:** Hüseyin Emre Tepedelenli̇oğlu, Mustafa Onur Karaca, Şefik Murat Arikan, Tolga Tolunay, Güray Toğral, Tezer Kutluk

**Affiliations:** 1 Department of Orthopedics and Traumatology, Dışkapı Yıldırım Beyazıt Eğitim ve Araştırma Hastanesi: Saglik Bilimleri Universitesi Diskapi Yildirim Beyazit Egitim ve Arastirma Hastanesi, Ankara, Türkiye; 2 Department of Orthopedics and Traumatology, Faculty of Medicine, Ankara University, Ankara, Türkiye Turkey; 3 Department of Orthopedics and Traumatology, Faculty of Medicine, Gazi University, Ankara, Türkiye Turkey; 4 Department of Orthopedics and Traumatology, Faculty of Medicine, Medipol University, Ankara, Türkiye Turkey; 5 Department of Orthopedics and Traumatology, Abdurrahman Yurtaslan Training and Research Hospital, Ankara, Türkiye Turkey; 6 Department of Pediatric Oncology, Faculty of Medicine, Hacettepe University, Ankara, Türkiye Turkey

**Keywords:** Infantile myofibromatosis, Extremity, β-catenin, Recurrence

## Abstract

**Background:**

Infantile myofibromatosis (IM) is a rare benign myofibroblastic neoplasm of infancy that usually involves the skin, bone, muscle, and soft tissue and rarely visceral organs. Questions/purposes: The aim of this study is to describe solitary IM involving bone or soft tissue in the extremities across different ages and sexes and to explore whether larger tumor size and beta-catenin (β-catenin) positivity were more frequently observed in recurrent cases.

**Methods:**

This multi-center study included 15 patients diagnosed and treated with IM between January 2004 and December 2019. All patients were diagnosed with incisional biopsy before definitive surgery to rule out the risk of sarcoma. Data including age, sex, duration of complaints, time to diagnosis, size, tumor histology, surgery type, complications, recurrence, functional outcomes and follow-up were recorded. β-catenin immunoreactivity was assessed in all patients, and the findings were analyzed descriptively because of the small sample size.

**Results:**

The mean age of the patients was 8.3 ± 6.4 years with male predominance. Recurrence was observed in five patients. Tumors larger than 5 cm and β-catenin positivity were more frequently observed in recurrent cases. Neurovascular involvement, documented on preoperative imaging and/or intraoperative findings, was seen in both recurrent and non-recurrent cases. Given the very small cohort, these observations are presented descriptively.

**Conclusion:**

Soft tissue IM is difficult to diagnose and can be misdiagnosed as malignancy. Therefore, biopsy before definitive surgery remains crucial. In this series, recurrence was observed in a subset of solitary extremity IM cases, and recurrent cases more often showed larger tumor size and β-catenin positivity. However; Further large-scale, prospective studies are needed to draw more reliable conclusions on this subject.

**Level of evidence:**

Level III, retrospective study.

**Clinical trial number:**

Not applicable.

## Introduction

Infantile myofibromatosis (IM) is a rare benign myofibroblastic neoplasm of infancy and childhood, also the most common fibrous tumor of infancy. It may involve the skin, bone, muscle, soft tissue and rarely visceral organs. It was first described by Williams and Schrum in 1951 [1]. The incidence of IM is extremely low, with approximately 1 in 150,000 individuals. Although the histology is benign, the clinical presentation can be alarming, particularly when lesions are deep, rapidly enlarging, or associated with osseous destruction.

To date, three different forms of IM have been defined: solitary, multicentric without visceral involvement, and multicentric with visceral involvement (also known as generalized) [2]. The solitary form is the most frequent and usually has an excellent prognosis, whereas generalized disease is associated with more aggressive behavior because of visceral involvement.

The solitary form tends to occur predominately in males and is typically identified in the dermis, subcutis or deep soft tissues. The most commonly affected parts of the body are head, the neck, the trunk, and very extremely rarely the limbs [3]. The extremities are distinctly uncommon sites for solitary IM. When these lesions arise in bone or deep soft tissue, they may mimic sarcoma, osteomyelitis, septic arthritis, or other tumor-like musculoskeletal conditions on clinical examination and imaging. For this reason, diagnosis often depends on tissue sampling and clinicopathological correlation rather than imaging alone.

Beta-catenin (β-catenin) represents a pivotal protein within the Wingless/Int-1 (Wnt) signaling pathway. It plays a crucial role in the regulation and coordination of cell-cell adhesion and gene transcriptional regulation, thereby influencing vital cell functions such as proliferation, apoptosis, migration, and differentiation. Interest in β-catenin expression in pediatric fibroblastic and myofibroblastic lesions has increased; however, its relevance in IM, particularly with respect to recurrence, remains uncertain [4].

In recent years, there is growing interest regarding the relationship between β-catenin and IM. Several studies have demonstrated that β-catenin plays a critical role in many signaling pathways that are crucial for cell growth, cell differentiation, and tissue regeneration. Although the exact relationship has not been fully elucidated yet, the pathogenesis is thought to involve abnormal fibroblast and myofibroblast proliferation in solitary IM [5].

In the literature, there are many reports on solitary IM affecting the head, neck or trunk. However, there is a limited number of reports regarding solitary IM affecting the extremities. Therefore in the present study, we aimed to evaluate the clinical and pathological spectrum of these rare lesions and to describe recurrence patterns, with particular attention to lesion size and β-catenin expression.

## Materials and methods

### Study design and study population

This multi-center, retrospective study was conducted at the Departments of four tertiary referral centers between January 2004 to December 2019. Prior to study, a written informed consent was obtained from each parent and/or legal guardians of the patients. The study was approved by the Gazi University, Faculty of Medicine, Ethics Committee (No: 2023 − 975 and Approval Date: 27/07/2023) and conducted in accordance with the principles of the Declaration of Helsinki.

Initially, a total of 87 patients were screened. Inclusion criteria were as follows: having a history as a diagnosis of IM. Exclusion criteria were as follows: IM not occurring as a solitary form (including multicentric and generalized types) and IM affecting body regions other than the extremities. Finally, 15 patients diagnosed and treated with IM were recruited. All patients were diagnosed with incisional biopsy before definitive surgery to rule out the risk of soft tissue sarcoma.

### Data collection and data analysis

Data including age, sex, duration of complaints, time to diagnosis, size, tumor histology, surgery type, complications, recurrence and functional outcomes, and follow-up were recorded.

### Imaging studies and immunohistochemistry

Radiography, ultrasonography (USG), and magnetic resonance imaging (MRI) were performed prior to diagnosis. To rule out sarcoma/malignancy, insertional biopsy was performed in all cases except for 2 patients with bone-localized involvement. Histopathological examination included hematoxylin and eosin (H&E) staining and immunohistochemistry for β-catenin, CD34, actin, desmin, S-100, CD31, caldesmon, factor 13 A, FLI-1, D2-40, ERG, muscle-specific actin (MSA), smooth muscle actin (SMA) and vimentin. In addition, tumor size, the surgical margins of the tumor and the presence of mitosis were examined. After the diagnosis of IM, positron emission tomography/computed tomography (PET/CT) and whole-body MRI with USG scanning were performed, considering the possibility of multicentric or generalized IM. Patients without additional foci were diagnosed with solitary IM and followed.

### Definitions

Magnetic resonance imaging scans were performed at regular intervals (every six months for the first year and annually thereafter) to assess the risk of recurrence in patients who were followed on a regular basis. Patients with suspicious lesions underwent an additional biopsy. Those with a pathological diagnosis of IM were classified as having a recurrence, and the tumor was excised through wide resection during reoperation. Postoperative follow-up was continued in the same manner. Patients with recurrence were compared to those without recurrence based on the aforementioned parameters.

### Statistical analysis

Statistical analysis was performed using the SPSS version 25.0 software (IBM Corp., Armonk, NY, USA). The Shapiro-Wilk test was used to determine whether variables were normally distributed. Continuous data were presented in mean ± standard deviation (SD) or median and interquartile range (IQR), while categorical data were presented in number and frequency. Logistic regression was attempted for age, size, and beta-catenin values. However, due to the insufficient number of cases for logistic regression and the fact that beta-catenin and size values were categorical rather than numerical variables, no significant results could be obtained. Thus, we did not consider incorporating regression into our analysis. A p value of < 0.05 was considered statistically significant.

## Results

Of the patients included in the study, 11 were males and four were females with a mean age of 8.3 ± 6.4 years (range, 3 months-15 years). Demographic and baseline characteristics of the patients are presented in Table 1.

**Table 1:** Demographic and baseline characteristics of patients (*n* = 15)

		*N*/% or mean ± SD
Age (months) (mean ± SD)		99.47 ± 76.55
Gender (n/%)	Female	4 (26,7)
	Male	11 (73,3)
Side of mass (n/%)	Right	7 (46,7)
	Left	8 (53,3)
Location of mass (n/%)	Cruris	3 (20,0)
	Femoral	3 (20,0)
	Hand	2 (13,3)
	Plantar foot	2 (13,3)
	Clavicle	1 (6,7)
	Foot	1 (6,7)
	Forehand	1 (6,7)
	Gluteus	1 (6,7)
	Tibia	1 (6,7)
Size of mass	< 5 cm	10 (66,7)
	5–10 cm	4 (26,7)
	> 10 cm	1 (6,7)
Type of resection (n/%)	Wide resection	13 (86,7)
	Curettage	2 (13,3)
Presence of invasion (n/%)	Present	3 (20,0)
	Non-present	17 (80,0)
Recurrence (n/%)	Yes	5 (33,3)
	No	10 (66,7)

SD: Standard deviation

Pre-, intra-, and postoperative data of the patients are summarized in Table 2. Among the five patients with recurrence, tumors larger than 5 cm were more common than in patients without recurrence. Neurovascular involvement, based on preoperative imaging and/or intraoperative findings, was observed in both recurrent and non-recurrent cases. Five cases with recurrence and one case without recurrence had β-catenin positivity. Given the small sample size, these findings are reported descriptively and should be interpreted with caution.

**Table 2:** Pre-, intra-, and postoperative data of patients (*n* = 15)

Patient No	Onset	Gender	Site	Location	Tumor Size	Surgery Type	Pathology	Recurrence	Outcome/Follow-up (Years)
*1* (Fig. 1, Fig. 2)	2 years	Male	L	Posterior lower leg	5 × 3,7 × 5,5 cm	Wide resection (With vascular reconstruction)	β-Catenin+, CD34+, Actin+, Desmin-	+ 3 Months	Revision-No lesion/7
*2*	4 years	Male	L	Gluteal region	10 × 5,5 × 5 cm	Wide resection (With vascular reconstruction)	Desmin+, Actin+, S- 100, CD31, margin-	-	No lesion/7
*3*	15 years	Female	R	Femoral Posterior	6,5 × 5 × 5 cm	Wide resection	β-Catenin+, Actin+, Desmin-, CD34-, S100-	+ 3 Months	Revision-No lesion/7
*4*	16 years	Female	R	Plantar Foot	1 × 0,8 × 0,5 cm	Wide resection	Mitosis-	-	No lesion/7
*5*	8 years	Female	L	Plantar Foot	1 × 1 × 1 cm	Wide resection	β-Catenin+	-	No lesion/8
*6*	4 years	Male	R	Clavicle	0,5 × 0,5 × 0,5 cm	Curettage+graft	β-Catenin-	-	No lesion/8
*7*	2 years	Male	L	Hand (Thenar)	1,5 × 1,4 × 1 cm	Wide resection	Actin+, Desmin-, CD34-, Caldesmon-, S100-, margin-	-	No lesion/7
*8*	10 years	Male	R	Hand (4th Finger)	2 × 0,6 × 0,6 cm	Wide resection	Desmin+, SMA-, S100-, Factor13A-, CD34-, β-Catenin-	-	No lesion/6
*9*	12 years	Male	R	Anterior lower leg	3,5 × 2 × 0,5 cm	Wide resection	β-Catenin+	+ 3 Months	Revision-No lesion/6
*10*	3 years	Female	R	2nd webspace of foot	11 × 10 × 6 mm	Wide resection	Desmin+, focal staining with actin, β-Catenin+, margin	+ 6 Months Neoadjuvant CT-	Revision-No lesion/9
*11*	3 years	Male	L	Femoral Anterior	14 × 7 × 6 cm	Wide resection	β-Catenin+	+ 3 years	Revision+meloxicam- Second recurrence- Observation-Remission/12
*12*	3 months	Male	L	Proximal lower arm	(28 × 17 × 15 mm)	Wide resection (With nerve reconstruction)	SMA+, MSA-, desmin, S-100, β-catenin-, CD31-, FLI-1, D2-40, ERG-	-	No lesion/4
*13*	5 months	Male	L	Gastrocnemius	6 × 5 × 3 cm	Wide resection	MSA+, SMA+, vimentin+	-	No lesion/19
*14*	2 years	Male	L	Femoral	3 × 4 × 3 cm	Wide resection	IM	-	No lesion/16
*15*	3 years	Male	R	Proximal Tibia	7 × 4 × 8 mm	Curettage+graft	MSA+, SMA+, Vimentin+, S100-, Factor 13 A-, CD31-, CD34-	-	No lesion/16

IM: Infantile myofibromatosis

The overall recurrence rate was 33.3% (5/15). Among the five patients with recurrence, one experienced a second recurrence (re-recurrence rate among recurrent cases, 20.0%; 1/5). Three patients developed recurrence at three months and had no further recurrence after revision surgery. One patient developed recurrence at six months and received four cycles of vincristine, actinomycin D, and cyclophosphamide (VAC) chemotherapy followed by repeat wide resection, with no further recurrence. One patient with obesity developed recurrence three years after wide resection. A second wide resection was performed and meloxicam was administered. One year later, the lesion recurred again, and vincristine, actinomycin D, and cyclophosphamide (VAC) chemotherapy was recommended; however, the parents declined treatment. During follow-up, the patient underwent a strict nutritional program, lost more than 20 kg over two years, and spontaneous regression was observed. The lesion was localized in bone in two patients and in soft tissue in the remaining 13 patients.

Microscopic examination and immunohistochemistry were routinely performed for the tumors obtained from all patients. The rates of positive staining for tumor markers were as follows: β-catenin: 40.0% (8/15), actin: 40.0% (6/15), SMA: 26.7% (4/15), MSA: 20.0% (3/15), vimentin: 20.0% (3/15), desmin, CD31, CD34, and S100: 6.7% (1/15).

## Discussion

Infantile myofibromatosis, a fibrous tumor of infancy and childhood, is characterized by the formation of nodular lesions affecting the skin, visceral organs, or bones. Patients may present with either a solitary or multicentric form. Although histopathological characteristics of both types are similar, clinical presentation and prognosis are different [6].

Bone involvement in the solitary type is around 5% and is mainly seen in the craniofacial bones. However, bone involvement in extremities is extremely rare. In the literature, there are some case reports regarding solitary intraosseous IM (Table 3). The majority of these case reports exhibit a solitary presentation, multicentric involvement, or a predilection for the head and neck region along with the extremities. To the best of our knowledge, our study is the most comprehensive study regarding solitary IM of the extremities. In the literature, one case of clavicle has been reported by Imaizumi et al., [7] one case of distal tibia by Inwards et al., [8] two cases of ulna and humerus by Wu et al. [9], one case of femur by Yamamoto et al., [10] and one case of distal ulna by Kindblom and Angervall [11]. In our case series, intraosseous IM was localized in the proximal tibia and clavicle and treated with curettage.

**Table 3:** Review of the literature

Authors	Year	Case Number	Type	Location	Method	Conclusion
Chung et al. (1)	1981	61	Solitary & multicentric	Various	Mixed	Recurrence/regression/death
Katz el. Al. (30)	1983	1	Solitary	Back	Resection	No recurrence
Pujol et al. (31)	1987	1	Solitary	Scalp	Resection	No recurrence
Matthews et al. (32)	1990	1	Solitary	Mandible	Resection	No recurrence
Beyer et al. (33)	1990	1	Solitary	Sacrum	Curettage	No recurrence
Inwards et al. (8)	1991	14	Solitary (Bone)	Head & neck	Resection	No recurrence
Hutchinson et al. (34)	1991	1	Solitary	Temporal	Resection	No recurrence
Agertoft el. al. (35)	1991	1	Solitary	Inguinal	Resection	No recurrence
Vigneswaran et al. (36)	1992	3	Solitary	Mandible	Curettage	No recurrence
Davies et al. (37)	1994	4	Multicentric	Visceral	Resection	No recurrence
Sonoda et al. (38)	1994	2	Solitary	Knee (Skin)	Conservative	Persistence/Regression
Damotte et al. (39)	1996	1	Solitary	Tibia	Curettage	No recurrence
Lo et. al. (40)	1997	1	Solitary	Upper lip	Conservative	Regression after 3 years
Niimura et al. (41)	1997	1	Solitary	Temporal	Curettage	No recurrence
Wada et al. (42)	1998	1	Solitary	Spinal canal	Resection	No recurrence
Kubota et al. (43)	1999	1	Solitary	Triceps	Resection	No recurrence
Imaizumi et al. (7)	1999	1	Solitary	Clavicle	Curettage	No recurrence
Ijima et al. (44)	1999	1	Solitary	Skin	Conservative	Regression after 20 months
Okamoto et al. (45)	2000	1	Solitary	Subcutaneous	Resection	No recurrence
Netscher et al. (46)	2001	1	Solitary	Hand	Resection (Twice)	No recurrence
Orozco-Covarrubias et al. (47)	2002	1	Multicentric	Skin	Conservative	Regression after 3 years
Ang et al. (48)	2004	1	Solitary	Skin	Resection	No recurrence
Petit et al. (49)	2004	1	Solitary	Skin	Resection	No recurrence
Weinberger et al. (50)	2007	1	Solitary	Left 2th finger	Resection	No recurrence
Youssef (51)	2009	1	Solitary	Lower leg	Resection	Recurrence/Anputation
Larralde et al. (52)	2010	9	Solitary & multicentric	Skin	Conservative/Resection	Mixed
Mynatt et al. (53)	2011	1	Solitary	Orbita	Conservative	Regression after 12 months
Mashiah et al. (54)	2014	28	Solitary & multicentric	Various	Resection/Conservative	Recurrence/regression/death
Al-Qattan et al. (55)	2016	6	Solitary	Hand	Resection	No recurrence
Larralde et al. (56)	2017	1	Multicentric	Various	Chemotherapy	Mixed
Salerno et al. (57)	2017	1	Multicentric	Various	Conservative	Regression after 1 year
Zhao et al. (58)	2020	32	Solitary	Various	Resection	2 cases recurred
Ren et al. (59)	2020	1	Solitary	Mandible	Curettage	No recurrence
Najwa et al. (60)	2023	1	Solitary	Lower arm	Resection	No recurrence
El Ouazzani et al. (61)	2023	1	Solitary	Temporal	Resection + CT	No recurrence

**Fig. 1:**
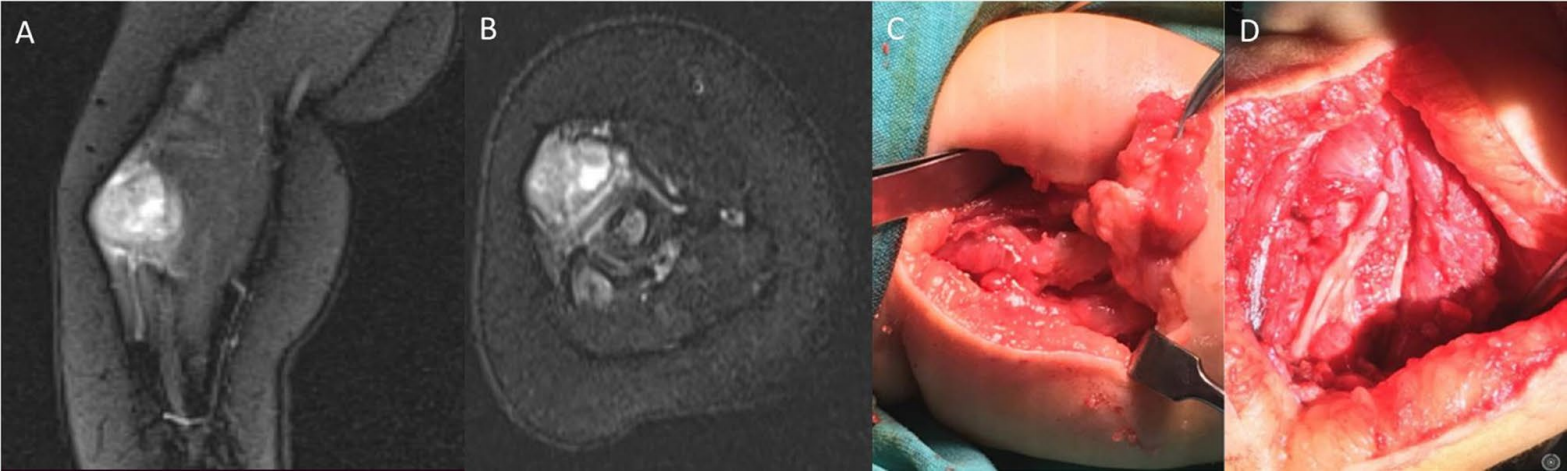
A 5-month-old male patient with a 28 × 17 × 15-mm solid mass in left proximal forearm **A**: T2 sagittal and **B**: T2 axial section, **C**: During wide resection, posterior 52. interosseous nerve (PIN) was found to be ruptured by the tumor D: After wide resection, was reconstructed with sural nerve graft

Infantile myofibromatosis has no specific cardinal symptoms. It is usually painless and appears as a mass visible to the naked eye. Intraosseous IM may present with pain due to the cystic nature or can be diagnosed incidentally. However, in soft tissues, IM can also cause neuropathy or vascular compression symptoms. If there is neurovascular involvement, depending on the neural intactness, nerve reconstruction with sural nerve graft may be needed. [12]. One of our patients had a non-iatrogenic posterior interosseous nerve rupture, resulting from the compression of the tumor (Fig. 1).

Furthermore, IM lacks distinct imaging characteristics. Radiographic imaging is primarily useful for intraosseous cases, which are often misdiagnosed as common benign bone lesions. Ultrasonography is useful to differentiate the lesion from cysts. The MRI imaging can reveal low intensity at T1 and high intensity of T2 sections. However, these findings are not specific, and lesions with irregular margins may mimic soft tissue sarcomas. IM may be misdiagnosed with desmoplastic fibroma, nodular fasciitis, peripheral nerve sheath tumors, hemangiopericytoma, leiomyoma, fibrosarcoma, and other spindle-cell lesions. In bone or juxta-articular lesions of the extremities, more common pediatric entities such as osteomyelitis, septic arthritis, eosinophilic granuloma, and other tumor-like lesions should also be considered [13, 14]. Therefore, a biopsy is recommended to rule out soft tissue sarcomas in the differential diagnosis [15]. In our study, we performed incisional biopsy in all 13 patients with soft tissue lesions before definitive surgery.

**Fig. 2:**
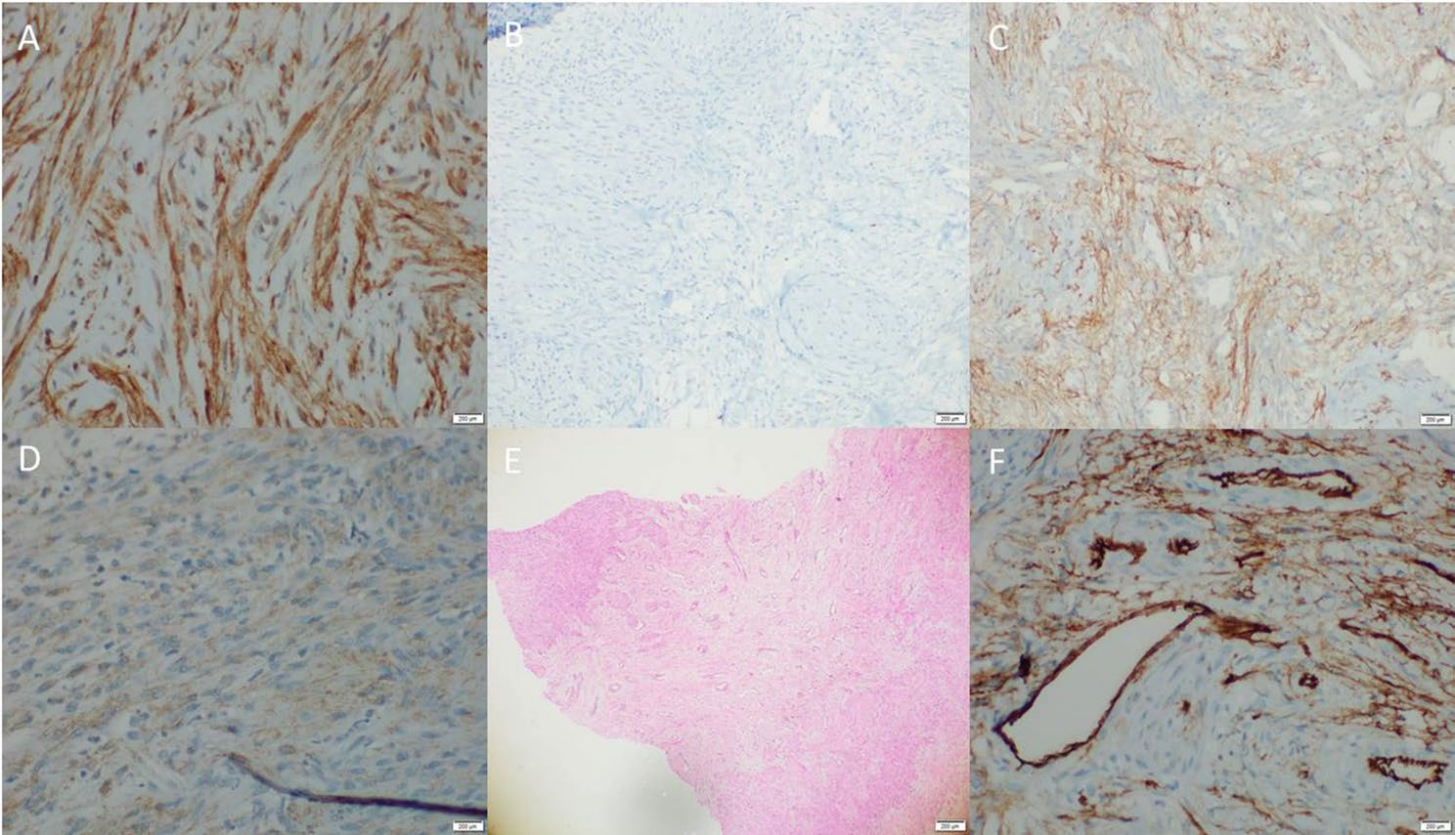
Patient 1. Immunohistological staining with **A**: MSA (100x magnification), **B**: Desmin (100x magnification), **C**: S-100 (100x magnification), **D**: SMA (100x magnification), **E**: Hematoxylin-eosin (100x magnification), **F**: CD34 (100x magnification)

Histologically, the cells exert a typical spindle-shaped fibroblastic or myofibroblastic appearance with a pink cytoplasm. The mitotic activity is slightly increased [16]. Immunohistological staining typically shows positivity for actin (smooth muscle SMA or muscle-specific MSA). β-catenin and vimentin positivity can be also obtained. However, staining usually produces negative results for CD31 and S-100 [17]. Desmin can be either positive or negative (Fig. 2). In our case series, we found vimentin positivity in two patients, S-100 negativity in five patients, CD31 negativity in three patients, desmin positivity in three patients, desmin negativity in four patients, and MSA positivity in three patients, consistent with the literature.

Solitary IM is usually seen on the skin and present at birth, and tends to regress spontaneously. On the contrary, soft tissue or intraosseous forms cannot be detected at birth, as they cannot be seen to the naked eye. Therefore, when a lesion is visible before the diagnosis is made, it is often confused with other soft tissue tumors. In most cases, IM is misdiagnosed as benign tumors as desmoplastic fibroma, schwannoma, hemangiopericytoma, leiomyoma, neurofibroma and malignant tumors such as leiomyosarcoma, fibrosarcoma, and neuroblastoma [17]. The main reason for misdiagnosis as malignancy is attributed to the presence of necrosis and vascular component in the tumor and pleomorphism.

After the diagnosis, whole-body scan is essential for the presence of visceral or multicentric form of IM. While the prognosis of visceral and multicentric IM is poor, solitary IM has usually excellent prognosis. The recurrence rate varies between 7 and 31%, depending on the tumor location and resection status (complete versus incomplete) [18]. Green et al. [17] reported an unusually aggressive case of the axilla which consequently required forequarter amputation with neck dissection and free-flap myocutaneous reconstruction was performed. The key to the treatment of IM is always to make biopsy first and then investigation for concomitant visceral involvement. For the solitary form of IM, total excision is the preferred technique. In the literature, there are case reports showing spontaneous regression [19]. Similarly, one of our cases also had a spontaneous regression after weight loss.

Infantile myofibromatosis usually occurs before the first two years of life. However, it is not uncommon to be seen at older ages, extending to early adulthood. Smith et al. [20] studied 34 adult cases with a median age of 36 years. Daimaru et al. [21] reported five solitary cases with a mean age of 48 years, two of which involved the upper and two of which involved the lower extremities. In their study, Beham et al. [16] published adult form of IM and their oldest case was 64 years old.

Solitary IM is a rare benign mesenchymal neoplasm. Unlike superficial dermatological lesions, musculoskeletal IM is usually managed with surgical excision. However, recurrence may occur after the initial excision and, in occasional cases, more than once. Thus, close follow-up is required, and parents and/or caregivers should be informed about the prognosis and the possibility of recurrence.In our series, lesions measuring ≥5 cm were more frequently observed among recurrent cases; however, this observation should be interpreted descriptively.

Although the exact etiology of IM still remains unclear, numerous hypotheses have been proposed to date. In some cases, IM can be congenital and inherited in both autosomal dominant and recessive patterns. Therefore, a detailed medical history should be obtained from the other members of the family and a thorough physical examination should be performed [22]. In our case series, no cases were reported or diagnosed in the family history. Furthermore, estrogen has been shown to be influential on IM development [23]. Although maternal estrogen is the main culprit, body estrogen may also affect the prognosis of IM. It is well established that estrogen is produced from adipose tissue, and its levels have been shown to increase in the case of obesity [23]. One of our cases had a history of obesity and had recurrence twice. More intriguingly, when the patient had a dramatic weight loss (over 20 kg), spontaneous regression was observed.

Moreover, platelet-derived growth factor receptor-beta (PDGFRB) gene variants have been discovered with autosomal-dominantly inherited IM [24]. On the other hand, PDGFRB variant may exist in sporadic cases, as well [25, 26]. Imatinib is a promising agent for the treatment of IM with PDGFRB mutation [25]. Another gene is NOTCH3. Besides, the role of other growth regulators such as basic fibroblast growth factor (bFGF) has been proposed in the pathogenesis of IM. It has been thought to induce angiogenic stimulation and regression [27].

The role of β-catenin positivity in the recurrence of IM is not well-established. β-catenin is a protein involved in cell adhesion and gene transcription, and its abnormal expression has been implicated in various tumors. However, specific studies linking β-catenin positivity to recurrence rates in IM are still lacking. Some research has indicated that mesoblastic nephroma, a tumor with histological similarities to IM, exhibits β-catenin expression, suggesting a potential, but not definitive link [28]. In another study, the relationship between pediatric fibrobastic and myofibroblastic lesions and beta-catenin was examined, and it was noted that beta-catenin expression was unexpected in common pediatric fibrobastic lesions, being more prevalent in adult-type myofibroblastic neoplasms [29]. In our study, β-catenin positivity was observed more often in recurrent cases; however, given the small sample size, this finding should be considered exploratory rather than confirmatory. At present, β-catenin expression may be viewed as a potential marker that deserves further evaluation in larger series.

Nonetheless, there are some limitations to this study. First, the number of patients was small due to the rarity of the case and its type. As a result, this type of rare pathology was collected from four centers which may have caused heterogeneity. Second, not all histopathological examinations could be conducted on every pathological specimen. Finally, after the cases were identified as solitary lesions through screening, PDGFRB gene analysis was unable to be performed due to various reasons. Further large-scale, well-designed studies are needed to draw more reliable conclusions on this subject.

## Conclusion

In conclusion, solitary IM of the extremities is a rare benign lesion that may mimic malignant or other tumor-like/inflammatory conditions, particularly when involving soft tissue or bone. Biopsy remains important for establishing the diagnosis before definitive surgery in soft tissue lesions. In this small retrospective series, recurrence was observed in a subset of patients, and recurrent cases more often showed larger size and β-catenin positivity. These observations should be interpreted cautiously as descriptive, hypothesis-generating findings rather than evidence of causation.

## Data Availability

The authors confirm that the data supporting the findings of this study are. available within the article and its Supplementary material. Raw data that support the findings of this study are available from the corresponding author, upon reasonable request.
